# Orbital Lesions: A bird’s eye view of series of 2068 cases in 27 years in a tertiary care hospital in Pakistan

**DOI:** 10.12669/pjms.40.8.9843

**Published:** 2024-09

**Authors:** Asad Aslam Khan, Sidrah Latif, Muhammad Ismail A Khan, Imran Ahmad

**Affiliations:** 1 Asad Aslam Khan, MBBS, MS, FCPS (Ban), Fellowship in Paediatric Ophthalmology, PhD Professor Emeritus, Institute of Ophthalmology, King Edward Medical University, Mayo Hospital Lahore, Pakistan; 2 Sidrah Latif, MBBS, FCPS, FRCOphth, MRCSEdin, FICO Assistant Professor, Services Institute of Medical Sciences, Lahore, Pakistan; 3 Muhammad Ismael Khan, Final Year MD Student St Martinus University, Medical School, Willemstad, Curacao; 4 Imran Ahmad, MBBS, DOMS, MPH. Principal Medical Officer, Institute of Ophthalmology, King Edward Medical University, Mayo Hospital Lahore, Pakistan

**Keywords:** Neoplastic lesions, Primary orbital lesions, Secondary orbital lesions, Hematopoietic orbital lesions, Metastatic orbital lesions

## Abstract

**Objective:**

To determine the relative frequency of orbital lesions based on the site of origin and histopathology at a Tertiary care hospital (Mayo Hospital, Lahore Pakistan) from 1996 till 2022 (27 years).

**Methods:**

This descriptive case series included 2651 patients of all age groups presenting with orbital lesions who initially got enrolled at Institute of Ophthalmology Mayo Hospital, Lahore from 1996 till 2022. Of these, 583 patients left against medical advice. So, clinical data of 2068 patients were completely analyzed. Lesions were managed medically and/ or surgically. Final clinical diagnosis, with the help of histopathology, was used to classify the lesions.

**Results:**

There were 1258 (60.9%) adults and 810 (39.1%) children, 1358 (65.66%) were neoplastic while 710 (34.33%) non-neoplastic lesions. Amongst the neoplastic lesions, 405 (29.8 %) were benign and 953 (70.2%) malignant. Primary orbital lesions were 1676 (81.04%), Secondary orbital lesions were 300 (14.51%), Endocrine/ hematopoietic reticulo-endothelial system lesions were 84 (4.06%) and Metastatic lesions from distant organs were 08 (0.39%).

**Conclusion:**

Retinoblastoma, rhabdomyosarcoma, optic nerve gliomata were common in children. Pleomorphic adenoma & adenocystic carcinoma of lacrimal gland, cavernous hemangioma, optic nerve meningioma, neurofibroma, schwannoma, squamous cell carcinoma of eyelid, carcinoma of maxillary antrum and lymphomas were more common in adults

## INTRODUCTION

Orbit is a pear-shaped paired cavity which contains tissues derived from all three germinal layers. Bones constituting orbital walls, eyeball, muscles, blood vessels, nerves and other orbital contents thus give rise to a wide variety of lesions.^1^ The lesions can be non-neoplastic as well as neoplastic, which can further be divided into benign or malignant lesions. Prevalence of tumors can show racial and geographical variations. A study carried out in Denmark shows the most common malignant tumor of the orbit to be Lymphoma, whereas a study in China indicates Malignant Lacrimal Gland tumor to be the commonest.^2^

There can be several effects related to masses in the orbit. Some lesions can remain harmless for years, whereas others can cause severe damage varying from loss of vision to loss of life, unless diagnosed and managed at an early stage. The reported frequency of the different types of orbital lesions varies greatly in the literature, which most likely is due to difference in the study population and age-group differences.^3^

In order to ascertain the disease burden for efficient strategic planning for their management, in this study different orbital lesions managed over a period of 27 years in all age groups have been analyzed. Frequency of different orbital lesions in children and adults who visited Institute of Ophthalmology, Mayo Hospital Lahore, Pakistan has been determined.

## METHODS

This descriptive case series study was done to determine the relative frequency of different types of orbital lesions presenting in a Tertiary Eye-care Hospital (Institute of Ophthalmology, Mayo Hospital, Lahore, Pakistan) during 27 years (from 1996 till 2022).

### Ethical Approval:

Registration of patients had started at a time when ethical approval was not considered mandatory. However, the same was obtained retrospectively from Ethical Review Board of College of Ophthalmology & Allied Vision Sciences, King Edward Medical University, Lahore) vide letter no. 1766/23, dated December 12, 2023.

Informed consent for data acquisition and management was obtained from the participants where possible. Out of the 2651 participants registered initially, 583 failed to follow-up, and their records could not be completed. We completed the analysis of remaining 2068 patients.

## RESULTS

Of the completely analyzed cases, 60.9% (n=1258) were adults and 39.1% (n=810) were children. About 56.08% (n=1160) were male patients and 43.92% (n=908) were female. 65.66% (n=1358) were neoplastic and 34.33% (n=710) non-neoplastic lesions. Amongst neoplastic 29.8% (n=405) were benign and 70.2% (n=953) malignant.

Primary orbital lesions (originating from any of the original orbital tissues) were 81.04% (n=1676), Secondary orbital lesions (invading the orbit secondarily after originating from surrounding paranasal sinuses, nasopharynx or cranial cavity) were 14.51% (n=300), Endocrine/Hematopoietic reticulo-endothelial system lesions were 4.06% (n=84) and Metastatic (where primary lesion is located elsewhere in the body) were 0.38% (n=08).

Amongst Primary Orbital lesions (Table-I), Ocular (> 90% of whom were retinoblastomas), and inflammatory lesions (mainly nonspecific chronic Inflammation, Fungal Granulomas, Pseudotumors, & Cellulitis) comprised more than half. Others were Cystic, Lacrimal gland lesions, Vascular, Optic Nerve Lesions, Muscular lesions, Nervous tissue related, Connective Tissue lesions and, Adipose Tissue derived lesions.

**Table-I:** Primary Orbital Tumors.

	Total	% of Primary	% of total
Primary orbital tumors	1676		81.04%
***Ocular***	482	28.76%	23.31%
•Retinoblastoma	440	26.25%	21.28%
•Malignant Melanoma	30	1.79%	1.45%
•Round blue cell tumor	12	0.72%	0.58%
***Inflammatory***	370	22.08%	17.89%
•CelluIitis	55	3.28%	2.66%
• Orbital abscess	20	1.19%	0.97%
• Sub-periosteal abscess	17	1.01%	0.82%
• Epidural Abscess	1	0.06%	0.05%
•Cavernous sinus	2	0.12%	0.10%
•Non-Specific	89	5.31%	4.30%
• Fungal Granuloma	71	4.24%	3.43%
•Pseudotumor	70	4.18%	3.38%
•Tuberculous	36	2.15%	1.74%
•Foreign Body Granuloma	5	0.30%	0.24%
•Giant Cell Granuloma	3	0.18%	0.15%
•Xantho-granulomatous	1	0.06%	0.05%
***Cystic***	247	14.74%	11.94%
•Dermoid Cyst	135	8.05%	6.53%
•Epidermal Cyst	33	1.97%	1.60%
•Simple Cyst	30	1.79%	1.45%
•Hydatid Cyst	10	0.60%	0.48%
•Hemorrhagic Cyst	7	0.42%	0.34%
•Chocolate Cyst	3	0.18%	0.15%
•Lipodermoid Cyst	11	0.66%	0.53%
•Limbal Dermoid	8	0.48%	0.39%
•Inflammatory Cysts	5	0.30%	0.24%
•Haematic Cyst	5	0.30%	0.24%
***Lacrimal Gland***	165	9.84%	7.98%
•Adenocystic CA	70	4.18%	3.38%
•Pleomorphic Adenoma	47	2.80%	2.27%
•Lymphomas	15	0.89%	0.73%
•Miscellaneous	7	0.42%	0.34%
•Tuberculosis	6	0.36%	0.29%
•Chronic dacryoadenitis	5	0.30%	0.24%
•Foreign Body	4	0.24%	0.19%
•Acute Inflammation	3	0.18%	0.15%
•Mikulics	2	0.12%	0.10%
•Acinic Cell Tumors	2	0.12%	0.10%
•Mucoepidermoid CA	2	0.12%	0.10%
•Lac Gland Abscess	1	0.06%	0.05%
•Lymphoepithelial	1	0.06%	0.05%
***Vascular***	145	8.65%	7.01%
•Cavernous Hemangioma	77	4.59%	3.72%
•Capillary Hemangioma	38	2.27%	1.84%
•Lymphangioma	10	0.60%	0.48%
•Orbital Varices	5	0.30%	0.24%
•A-V Malformation	5	0.30%	0.24%
•Malignant Hemiangiopericytoma	3	0.18%	0.15%
•Carotid Cavernous Fistula	2	0.12%	0.10%
•Pericytoma	1	0.06%	0.05%
•Cavernous Sinus Fistula	1	0.06%	0.05%
•Haemangioblastoma	1	0.06%	0.05%
•Malignant Angioma	1	0.06%	0.05%
•Glomus tumor	1	0.06%	0.05%
***Optic Nerve***	69	4.12%	3.34%
•Optic Nerve Glioma	35	2.09%	1.69%
•Meningioma	34	2.03%	1.64%
***Muscular***	62	3.70%	3.00%
•Rhabdomyosarcoma	57	3.40%	2.76%
•Myositis	5	0.30%	0.24%
***Nervous tissue***	57	3.40%	2.76%
•Neurofibroma	29	1.73%	1.40%
•Schwanoma	27	1.61%	1.31%
•Malignant peripheral nerve sheath tumor	1	0.06%	0.05%
***Connective tissue***	29	1.73%	1.40%
•Fibrous/histiocytoma	15	0.89%	0.73%
•Soft tissue sarcoma	12	0.72%	0.58%
•Hamartoma	2	0.12%	0.10%
•Ch Osteomyelitis	1	0.06%	0.05%
***Congenital lesion***	9	0.54%	0.44%
•Amniontocele	4	0.24%	0.19%
•Polycystic Eye	1	0.06%	0.05%
•Heterotropic Glial Tissue	1	0.06%	0.05%
•Craniosynostosis	1	0.06%	0.05%
•Mature Teratoma	1	0.06%	0.05%
•Corneal squamous Papilloma/ myxoid sarcoma	1	0.06%	0.05%

Cystic lesions included mainly Dermoid & epidermoid cysts, while most of the lacrimal gland lesions were Adeno-cystic carcinomas and pleomorphic adenoma. Cavernous and capillary hemangiomas made bulk of vascular tumors while meningioma and gliomas were optic nerve tumors. Rhabdomyosarcoma was almost exclusive among muscular tumors while Neurofibroma and Schwannoma represented nervous tissue. Fibrous/histiocytoma and soft tissue sarcoma were main connective tissue tumors, osteoma and osteogenic sarcoma were main osseous tumors. Congenital lesions like amniontocele were also rarely present.

Amongst 300 Secondary Orbital Lesions (Table-II), almost 2/3 were from Eyelids, and 1/3 from Paranasal Sinuses, while minor contribution from cranial cavity and Nasopharynx was seen. Squamous cell carcinoma was the main eyelid tumor followed by hemangioma, Basal Cell Carcinoma, Sebaceous Cell Carcinoma, Malignant Melanoma etc. Paranasal sinuses lesion included Chronic Ethmoiditis, frontal sinus Mucocele and pyocele, and maxillary sinus carcinoma. Encephalocele and meningocele were seen in the cranial cavity while a solitary case of nasopharyngeal fibroma was also seen.

**Table-II:** Secondary orbital tumors.

	Total	% of Secondary	% of total
Secondary orbital tumors	300		14.51%
Eyelid	197	65.67%	9.53%
•Squamous Cell Carcinoma	117	39.00%	5.66%
•Haemangioma	22	7.33%	1.06%
•Basal Cell Carcinoma	21	7.00%	1.02%
•Sebaceous Cell Carcinoma	19	6.33%	0.92%
•Malignant Melanoma	7	2.33%	0.34%
•Cyst of Moll	3	1.00%	0.15%
•Malignant Sebaceous Cyst	2	0.67%	0.10%
•Apocrine hydrocystoma	2	0.67%	0.10%
•Adenocarcinoma meibomian gland	2	0.67%	0.10%
•Trichoepithelioma	1	0.33%	0.05%
•Apocrine carcinoma	1	0.33%	0.05%
Paranasal sinuses	99	33.00%	4.79%
Ethmoidal	48	16.00%	2.32%
•Ch Ethmoiditis	44	14.67%	2.13%
•Inflammed Nasal Polyp	2	0.67%	0.10%
•Inverted Papilloma	1	0.33%	0.05%
•Ethmoidocele	1	0.33%	0.05%
Frontal	28	9.33%	1.35%
•Mucocele	19	6.33%	0.92%
•Pyocele	8	2.67%	0.39%
•Dacryocele	1	0.33%	0.05%
Maxillary	23	7.67%	1.11%
•Maxillary Antrum CA	20	6.67%	0.97%
•Ameloblastoma	2	0.67%	0.10%
•Maxillary polyp	1	0.33%	0.05%
Cranial cavity	3	1.00%	0.15%
•Encephalocoele	2	0.67%	0.10%
•Meningocoele	1	0.33%	0.05%
Nasopharynx	1	0.33%	0.05%
•Nasopharyngeal Angiofibroma	1	0.33%	0.05%

Amongst 84 Endocrine/Hematopoietic reticulo-endothelial system lesions (Table-III), Lymphomas/ Leukemias were predominant while Thyroid Eye Disease (TED), Angio-lymphoid hyperplasia and Plasmacytoma were also seen. Amongst 08 Metastatic lesions (Table-IV), Secondaries from Ewing Sarcoma of bone were found in 2/3 cases. However, in more than a third of the cases primary lesion could not be ascertained.

**Table-III:** Hematopoietic/ Reticulo-Endothelial/ Endocrine Tumors.

	Total	% of Hem/RE/Endo	% of total
Hematopoietic/ RE/ Endocrine	84		4.06%
•Lymphoma/ Leukemia	67	79.76%	3.24%
•Thyroid Eye disease	10	11.90%	0.48%
•Angiolymphoid hyperplasia	5	5.95%	0.24%
•Plasmacytoma	1	1.19%	0.05%
•Castleman Disease	1	1.19%	0.05%

**Table-IV:** Metastatic Tumors.

	Total	% of Metastatic	% of total
Metastatic	8		0.39%
•Ewing Sarcoma	5	62.50%	0.24%
•Unknown	3	37.50%	0.15%

## DISCUSSION

In the current study, 60.9% (1258) lesions were in adults and 39.1% (810) were in children, 65.66% (1358) were neoplastic and 34.33% (710) non-neoplastic lesions. Amongst neoplastic, 29.8% (405) were benign and 70.2% (953) malignant. These findings are similar to a recent study done in Iran which also found that benign lesions are found more commonly in children while malignant lesions are more prevalent in ages 60 & above.^4^ Other studies however suggest that 60-66% of orbital lesions are benign.^5^,^6^

Retinoblastoma was the most common primary orbital, ocular tumor in our series. According to Silvera, Retinoblastoma is commonest in children being diagnosed in one in 16000 children and 8000 to 10000 children being affected annually. Being highly malignant, an early diagnosis is very important for preserving life, eye and sight.^7^ Inflammatory, cystic, lacrimal and vascular tumors follow retinoblastoma as more common primary tumors in our series. In a study done on 1264 orbital tumors in the USA, major groups were similar to our series with some minor variations in prevalence. In the latter, vascular lesions were more common followed by inflammatory, lacrimal, optic nerve and meningeal, and cystic lesions.^8^

Secondary tumors arise from the eyelids and the surrounding structures like paranasal sinuses, cranial cavity etc.^9^,^10^ Squamous cell carcinoma was the most common in our series whereas Basal cell carcinoma is usually common in the West.^11^ However a study by Nageli et al. supports our findings.^10^ Paranasal sinuses can contribute towards spread of lesion into the orbit. These may be chronic inflammations leading to chronic ethmoiditis, Frontal mucocele/ pyocele, or neoplastic lesions like adenocarcinoma of the maxillary antrum. Mucocele of the sinuses are due to blockage of the ostia and chronic inflammations/ allergic processes.^12^ Build up of normal secretions from the goblet cells residing within the columnar epithelia of the sinuses is the pathogenetic mechanism of a mucocele formation.^13^

Like elsewhere, the orbit was found to be affected by hematopoietic/ Reticuloendothelial system/ Endocrine system with a prevalence of about 4%.^14^ These lesions were mainly lymphomas/ leukemias, thyroid eye disease and angiolymphoid hyperplasia. Metastatic orbital tumors are rare (1-13%) with primary lesion in breast, lung, colon, bone etc.^14^ In our series, the proportion of metastatic tumors was only 0.39% with primary arising from Ewing Sarcoma in 62.5% of the tumors, while in the rest primary site could not be ascertained despite best efforts.

The most common sign of orbital lesion is proptosis or forward displacement of the eyeball.^15^ The site of the lesion can determine the direction of displacement such as upward from maxillary sinus lesion or inferomedial from lacrimal gland tumors.^16^ Based on location in relationship to the extraocular muscles, the lesion can be described as intraconal or extraconal.^17^ Intraconal lesion like optic nerve tumors may produce vision loss, axial proptosis and restriction in extraocular movements with or without diplopia.

Extraconal lesions mainly present with displacement of eyeball and visual loss occurs late.^18^ Occasionally a palpable space occupying lesion (SOL) may be seen which could transform into a fungating mass if too much time has elapsed.^19^ Imaging techniques like CT Scan, MRI and ultrasonography (B scan) play an important role in localization, demarcation and sometimes, diagnosis of the tumor.^20^ Benign lesions usually show localization with well defined capsule or lining, whereas malignant lesions are mostly irregular, with no well defined margin and erosion of surrounding tissues.^21^ Surgery can be employed as a diagnostic tool (for histopathology) and also as a treatment. De-bulking of large masses can help followed by chemotherapy or radiation.^15^

### Limitations:

Ethical permission from Ethical Review Board could not be taken beforehand as the study started in 1996 when no such requirement existed.

## CONCLUSION

Orbital lesions are more common in adults as compared to children. Primary orbital lesions are the most common lesions. Secondary orbital lesions also pose a major disease burden. Patients visiting tertiary care hospital presented a wider cohort of neoplastic lesions with predominance of malignant neoplastic lesions. Tertiary care centers for Orbital diseases should have strong inter-departmental liaisons to provide excellent radiological, histopathological, oncological care within one premises along with orbital care.

### Authors Contribution:

**AAK:** Main Concept and design, interpretation of data, edited and approved the final manuscript and responsible for accuracy and integrity of the work.

**SL:** Concept and design, data cleaning, preparation of the manuscript.

**MIAK**: Concept and design, manuscript editing and interpretation of the data.

**IA:** Preparation, revision and editing of final manuscript, Statistical Analysis.
